# O Desafio de Tornar a Ressonância Cardíaca uma Realidade Global

**DOI:** 10.36660/abc.20230187

**Published:** 2023-04-12

**Authors:** Hélder Jorge Andrade Gomes, Alcides Rocha de Figueredo

**Affiliations:** 1 Departamento de Clínica Médica Faculdade de Medicina de Jundiaí Jundiaí SP Brasil Departamento de Clínica Médica – Faculdade de Medicina de Jundiaí, Jundiaí, SP – Brasil; 2 ICON Diagnósticos por Imagem Jundiaí SP Brasil Tomografia e Ressonância Cardiovascular – ICON Diagnósticos por Imagem, Jundiaí, SP – Brasil; 3 Hospital Vera Cruz Campinas SP Brasil Hospital Vera Cruz, Campinas, SP – Brasil; 4 Prevent Senior São Paulo SP Brasil Prevent Senior, São Paulo, SP – Brasil; 5 Hospital Samaritano de São Paulo São Paulo SP Brasil Hospital Samaritano de São Paulo, São Paulo, SP – Brasil

**Keywords:** Diagnóstico por Imagem/tendências, Espectroscopia de Ressonância Magnética/métodos, Doenças Cardiovasculares/mortalidade, Morte Súbita, Cardiomiopatas, Fibrose Endomiocárdica, Isquemia Miocárdica, Volume Sistólico

Apesar de redução substancial nas taxas de mortalidade cardiovascular ajustada por idade nas últimas décadas, a doença cardiovascular continua sendo a causa mais comum de morte.^1^ Estudos estimam que a morte súbita cardíaca seja responsável por aproximadamente 50% de todos os óbitos por causas cardiovasculares, sendo as taquicardias ventriculares com degeneração para fibrilação ventricular e assistolia a sequência fatal de eventos fisiopatológicos mais comum.^2^

O árduo trabalho realizado pelos autores do artigo^3^ publicado nos Arquivos Brasileiros de Cardiologia ilustra o papel da ressonância magnética cardíaca (RMC) para a elucidação etiológica dos eventos de morte súbita encontrados na prática clínica. Além das já esperadas doenças de Chagas e isquêmica, uma grande variedade de outros diagnósticos foram reconhecidos: miocardites, cardiomiopatia hipertrófica, cardiomiopatia dilatada, a cardiopatia arritmogênica, distrofia muscular, cardiomiopatia hipertensiva, metástases, miocárdio não compactado, e cardiomiopatia adrenérgica (Síndrome de Takotsubo). A RMC foi conclusiva em mais de 90% dos casos e é uma ferramenta chave para a exclusão de cardiopatia estrutural e atribuição do evento fatal a causas extracardíacas.

Por outro lado, o estudo mostrou o quanto estamos longe de bons critérios para prevenção de morte súbita cardíaca pela fração de ejeção do ventrículo esquerdo (FEVE): apenas um terço dos pacientes apresentavam uma FEVE ≤ 35%, e quase 40% dos indivíduos apresentavam uma FEVE > 50%. Não é de hoje que os estudos indicam que a FEVE por si só não é um critério suficiente para a identificação adequada dos pacientes que mais se beneficiam do CDI.^4^ Portanto, nos últimos anos, esforços têm sido feitos para identificar novos marcadores capazes de refinar a estratificação prognóstica da morte súbita. A análise genética e a ressonância magnética parecem ser ferramentas extremamente úteis para o aprimoramento desse processo.^5^

Recentemente, a Sociedade Brasileira de Cardiologia atualizou suas diretrizes sobre dispositivos cardíacos eletrônicos implantáveis^6^ e, embora tenha mantido a FEVE ≤ 35% como parâmetro principal para indicação de cardioversor-desfibrilador implantável (CDI) na prevenção primária de morte súbita, novas ferramentas foram incorporadas neste processo, em especial para as cardiopatias não-isquêmicas, como a detecção de fibrose pela RMC ou de alterações genéticas de alto risco por testes moleculares.

A fibrose miocárdica detectada pela RMC com a técnica de realce tardio com gadolínio esteve presente em aproximadamente 70% dos casos de morte súbita do presente estudo,^3^ e é de fato um forte preditor de arritmia ventricular e morte súbita, tanto na cardiopatia isquêmica quanto na não isquêmica.^7 - 11^ Especialmente na cardiopatia dilatada não isquêmica, a presença de realce tardio em pacientes com FEVE > 35% ofereceu maior risco arrítmico do que naqueles pacientes com FEVE 21-35% mas sem realce tardio, re-estratificando o risco, portanto, em um terço dos casos.^12^

A ressonância cardíaca é uma modalidade de imagem cardíaca multifunção, com uma grande variedade de informações, usada para avaliação de morfologia, função, caracterização tecidual, perfusão, fluxos e cicatrizes/fibroses. A pesquisa de isquemia miocárdica pela RMC tornou-se uma parte importante do arsenal de imagem cardíaca, pois demonstrou ser mais precisa do que a SPECT e oferecer excelentes informações prognósticas.^13 , 14^ Na avaliação das cardiomiopatias, pode ser uma técnica importante para poder excluir a doença arterial coronariana como causa básica. No entanto, estudos recentes sugerem que o realce tardio com gadolínio pode ser suficiente para estabelecer a etiologia da insuficiência cardíaca na maioria das vezes.^15^

Nos últimos anos presenciamos uma avalanche de artigos científicos de ressonância cardíaca sobre a utilidade dos mapas paramétricos, seja no acometimento cardíaco precoce de diferentes patologias ou no diagnóstico etiológico específico, especialmente do Mapa T1 no diagnóstico diferencial de fenótipos hipertróficos como a Amiloidose Cardíaca ou a Doença de Anderson Fabry.^16 - 18^No entanto, essas técnicas requerem software avançado, bem como tempo significativo para adquirir dados pré e pós-contraste, e o uso desta tecnologia em países em desenvolvimento é caro e consome tempo de aquisição e pós-processamento.

Mesmo com tanta possibilidade de informação, no entanto, a RMC ainda é pouco utilizada, principalmente devido à sua falta de disponibilidade, por ser uma técnica considerada complicada, lenta, de alto custo e, em alguns casos em que a compreensão da técnica é limitada, pode não agregar ao arsenal diagnóstico. Diante deste quadro, o estudo INCA^19^ realizado em Lima, no Peru, e posteriormente replicado em outros países em desenvolvimento,^20^ demonstrou ser possível realizar o exame em menos de 30 minutos, reduzindo em 30-60% seus custos, mas sem perder seu alto impacto no manejo das cardiopatias.

Neste sentido, o esforço pela implementação e difusão de protocolos mais rápidos de ressonância cardíaca aumentariam o acesso ao método, oferecendo informação precisa para intervenções capazes de reduzir a morbimortalidade cardiovascular. A ressonância cardíaca precisa ser mais rápida, fácil e econômica para entrega global e sustentável.
