# Supplementary material for: O Desafio de Tornar a Ressonância Cardíaca uma Realidade Global
Source: Arq Bras Cardiol. 2023 Apr 12;120(4):e20230187. [Article in Portuguese] doi: 10.36660/abc.20230187 (PMC10124572; doi:10.36660/abc.20230187)
Supplement: Translation

# Translation: The Challenge of Making Cardiac Resonance a Global Reality

**Authors:** Hélder Jorge Andrade Gomes, Alcides Rocha de Figueredo

**Affiliations:** 1 Brazil Departamento de Clínica Médica – Faculdade de Medicina de Jundiaí, Jundiaí, SP – Brazil; 2 Brazil Tomografia e Ressonância Cardiovascular – ICON Diagnósticos por Imagem, Jundiaí, SP – Brazil; 3 Brazil Hospital Vera Cruz, Campinas, SP – Brazil; 4 Brazil Prevent Senior, São Paulo, SP – Brazil; 5 Brazil Hospital Samaritano de São Paulo, São Paulo, SP – Brazil

**Keywords:** Diagnostic Imaging/trends, Magnetic Resonance Spectroscopy, Cardiovascular Diseases/mortality, Death,Sudden, Cardiomyopathes, Endomyocardial Fibrosis, Myocardial Ischemia, Stroke Volume

Despite substantially reducing age-adjusted cardiovascular mortality rates in recent decades, cardiovascular disease remains the most common cause of death.^1^Studies estimate that sudden cardiac death is responsible for approximately 50% of all deaths from cardiovascular causes, with ventricular tachycardia with degeneration to ventricular fibrillation and asystole being the most common fatal sequence of pathophysiological events.^2^

The hard work done by authors of the article^3^published in the *Arquivos Brasileiros de Cardiologia* illustrates the role of cardiac magnetic resonance (CMR) in the etiological elucidation of sudden death events encountered in clinical practice. In addition to the already expected Chagas and ischemic diseases, a wide variety of other diagnoses have been recognized: myocarditis, hypertrophic cardiomyopathy, dilated cardiomyopathy, arrhythmogenic heart disease, muscular dystrophy, hypertensive cardiomyopathy, metastases, non-compacted myocardium, and adrenergic cardiomyopathy (Takotsubo syndrome). The CMR was conclusive in more than 90% of cases and is a key tool for excluding structural heart disease and attributing the fatal event to extracardiac causes.

On the other hand, the study showed how far we are from good criteria for the prevention of sudden cardiac death by left ventricular ejection fraction (LVEF): only one-third of the patients had an LVEF ≤ 35%, and almost 40% of the individuals had an LVEF > 50%. It is not new that studies indicate that LVEF alone is not a sufficient criterion for the proper identification of patients whom most benefit from ICD.^4^Therefore, in recent years, efforts have been made to identify new markers capable of refining the prognostic stratification of sudden death. Genetic analysis and magnetic resonance imaging are extremely useful tools for improving this process.^5^

The Brazilian Society of Cardiology recently updated its guidelines on implantable electronic cardiac devices.^6^Although it maintained LVEF ≤ 35% as the main parameter for indicating an implantable cardioverter-defibrillator (ICD) in the primary prevention of sudden death, new tools were incorporated into this process, especially for non-ischemic heart diseases, such as the detection of fibrosis by CMR or high-risk genetic alterations by molecular tests.

Myocardial fibrosis detected by CMR with the delayed gadolinium enhancement technique was present in approximately 70% of the cases of sudden death in the present study^3^and is a strong predictor of ventricular arrhythmia and sudden death, both in ischemic and non-ischemic heart disease.^7 - 11^Especially in non-ischemic dilated heart disease, a delayed enhancement in patients with LVEF > 35% offered a higher arrhythmic risk than in those patients with LVEF 21-35% but without delayed enhancement, thus re-stratifying the risk in one-third of cases.^11^

Cardiac resonance is a multipurpose cardiac imaging modality with a wide variety of information used to assess morphology, function, tissue characterization, perfusion, flows, and scars/fibrosis. Myocardial ischemia research by CMR has become an important part of the cardiac imaging arsenal, as it is more accurate than SPECT and offers excellent prognostic information.^13 , 14^In evaluating cardiomyopathies, it can be an important technique to exclude coronary artery disease as the underlying cause. However, recent studies suggest that delayed gadolinium enhancement may be sufficient to establish the etiology of heart failure in most cases.^15^

In recent years, we have witnessed an avalanche of scientific articles on cardiac resonance imaging on the usefulness of parametric maps, either in the early cardiac involvement of different pathologies or in the specific etiological diagnosis, especially the T1 Map in the differential diagnosis of hypertrophic phenotypes such as Cardiac Amyloidosis or by Anderson Fabry.^16 , 18^However, these techniques require advanced software and significant time to acquire pre- and post-contrast data, and using this technology in developing countries is expensive and time-consuming acquisition and post-processing.

Even with so much possibility of information, however, CMR is still little used, mainly due to its lack of availability, as it is a technique considered complicated, slow, expensive, and, in some cases where the understanding of the technique is limited, may not add to the diagnostic arsenal. Because of this situation, the INCA^19^study carried out in Lima, Peru, and later replicated in other developing countries,^20^demonstrated that it is possible to perform the test in less than 30 minutes, reducing costs by 30-60%, but without losing its high impact in the management of heart disease.

In this sense, the effort to implement and disseminate faster cardiac resonance protocols would increase access to the method, offering accurate information for interventions capable of reducing cardiovascular morbidity and mortality. Cardiac MRI must be faster, easier, and more cost-effective for global and sustainable delivery.
